# Chitosan nanoparticle-delivered siRNA reduces *CXCR4* expression and sensitizes breast cancer cells to cisplatin

**DOI:** 10.1042/BSR20170122

**Published:** 2017-06-27

**Authors:** Shaonan Yu, Yan Chen, Xuefeng Li, Zhongli Gao, Guifeng Liu

**Affiliations:** 1 Department of Radiology, China-Japan Union Hospital of Jilin University, 130033 Changchun, China; 2 Department of Endocrinology, Second Hospital of Jilin University, 130041 Changchun, China; 3 Department of Anesthesiology, China-Japan Union Hospital of Jilin University, 130033 Changchun, China; 4 Department of Orthopedics, China-Japan Union Hospital of Jilin University, 130033 Changchun, China

**Keywords:** Breast cancer cells, Chitosan, Cisplatin, CXCR4, siRNA

## Abstract

Chemokine (C-X-C motif) receptor 4 (CXCR4) has been reported as a poor prognostic biomarker in human breast cancers, and has been suggested as a promising therapeutic target of breast cancer treatment. The present study aims to investigate the delivery efficiency of siRNA by chitosan into breast cancer cells, and then to examine the regulatory role by chitosan nanoparticle-delivered siRNA on *CXCR4* expression and on the chemosensitivity of breast cancer cells. Our results demonstrated that the siRNA could be capsuled by chitosan into nanoparticles with a diameter of 80–110 nm, and with a zeta potential of 20–50 mV. The chitosan nanoparticle delivered siRNA efficiently into breast cancer MCF-7 cells significantly reduced the expression of *CXCR4* in both mRNA and protein levels. Moreover, the reduced CXCR4 by chitosan nanoparticle-delivered siRNA was associated with increased sensitivity of breast cancer cells to cisplatin. Reduced growth and increased apoptosis of MCF-7 cells were observed in the CXCR4 siRNA group than in the control siRNA group. Taken together, our results present the treatment potential of chitosan nanoparticle-delivered siRNA targeting CXCR4 in breast cancers.

## Introduction

Breast cancer is the leading cause of cancer death in females worldwide. Approximately 1 of 8 women in western countries is affected by breast cancer, and 5% of which is caused by mutations in breast cancer associated gene 1 (*BRCA1*) and breast cancer associated gene 2 (*BRCA2*) [1,2]. Most of patients receive combined chemotherapy when diagnosed, but approximately 30% of these patients die of metastatic disease within 5 years [3]. Cisplatin is a conventional drug for breast cancer and for other solid tumors. It is a crosslink-inducing DNA-damaging agent that may also induce cell death by damaging cytoplasmic proteins and by inducing apoptosis at the execution phase level [4,5].

Patients with breast cancer often initially have a positive reaction to cisplatin-based chemotherapy. However, drug resistance is still a fundamental problem in breast cancer treatment, and is responsible for most treatment failures [6]. Cisplatin causes cytotoxicity to normal tissues, and cancer cells acquire resistance and reduce the drug’s toxicity [7]. The underlying mechanism of cisplatin resistance is not clear. Previous studies have indicated that the development of drug resistance is correlated with the tumor microenvironment, and chemokines also seem to play key roles in tumor progression and metastasis [8,9].

CXCR4 and its ligand CXCL12 (also named stromal cell-derived factor 1, SDF-1) have been shown to be consistently expressed in human breast cancer cells, and the activation of SDF-1α/CXCR4 axis has found to play a key role in breast cancer migration and metastasis [10–12]. It has been reported that CXCR4 is not only correlated with the metastatic spread of breast cancer cells, but also crucial in the tumor dissemination [13]. Thus, therapies targeting CXCR4 have recently attracted increased attention from researchers.

Here, we aimed to investigate the delivery efficiency of siRNA by chitosan into breast cancer cells, and then to examine the regulatory role by chitosan nanoparticle-delivered siRNA on *CXCR4* expression and on the chemosensitivity of breast cancer cells. We found that the siRNA could be capsuled by chitosan into nanoparticles efficiently. The chitosan nanoparticle delivered siRNA efficiently into breast cancer MCF-7 cells and significantly reduced the expression of *CXCR4* in both mRNA and protein levels. Additionally, the reduced CXCR4 by chitosan nanoparticle-delivered siRNA was associated with increased sensitivity of breast cancer cells to cisplatin.

## Materials and methods

### Cell culture

All cell culture reagents were ordered from Gibco (U.S.A.). Human breast cancer MCF‐7 cells were grown at 37°C in a 5% CO_2_ incubator in DMEM supplemented with L‐glutamine (2 mM), penicillin (100 U/ml), streptomycin (100 μg/ml), and 10% fetal calf serum (FCS).

### Chitosan nanoparticles preparation

Briefly, chitosan (114 KDa) was dissolved separately in acetate buffer (0.1 M sodium acetate/0.1 M acetic acid, pH 4.5) to form different concentrations of chitosan solution (25–300 μg/ml). Chitosan–siRNA complexes were produced by adding chitosan solution to an equal volume of siRNA solution (20 μg/ml) and quickly mixed, then the mixture was incubated at room temperature for 30 min to form chitosan–siRNA complexes.

### Zeta potential determination

Zeta potential measurements were performed using dynamic light scattering (Malvern Instruments, Malvern, U.K.) applying PALS zeta potential analyzer software as previously described [14]. The sample was run at least three times and each run lasted 50 cycles at 298 K with the Smoluchowski model. The zeta potential in millivolt units was calculated as the electrophoretic mobility (μm/cm[V·s]^−1^).

### siRNA transfection

The siRNA sequence was designed as previously reported [15]. Lipofectamine 2000 and chitosan nanoparticles were used for siRNA transfection respectively. For Lipofectamine 2000, transfection was performed as per standard protocol. Chitosan nanoparticle transfection was performed as previously described [16]. The cells were seeded in a 96**-**well plate at a density of 30,000 cells per well in Opti-MEM 1 reduced serum medium containing 5% of FBS without antibiotics, 24 h prior to transfection. On the day of transfection, 50 μl of chitosan–siRNA nanoparticles, siRNA alone, or Lipofectamine 2000–siRNA complexes (each well or formulation contained 4 pmol of siRNA) in the medium without serum was then added to the cells and incubated at 37°C with a 5% CO_2_ atmosphere for 48 h.

### Determination of nanoparticle morphology using transmission electron microscopy

Chitosan/siRNA nanoparticles were diluted 1/10 using 0.2 μm filtered sodium acetate buffer. A sample volume of 15 μl was immobilized onto freshly cleaved mica. The samples were purged with N_2_ and observed on FEI TECNAI 12 electron microscope with accelerating voltage of 80 KV. Several images were obtained for each sample, ensuring data reproducibility.

### Cy5-labeled siRNA chitosan nanoparticles in transfected cells

MCF-7 cells (85% confluence) were transfected with 100 nM siRNA using chitosan nanoparticles. The cells were transfected with the nanoparticles in serum-free DMEM for 1–4 h, after which 10% serum was added. Twenty four hours post transfection, cells were subjected to Hoechst staining to visualize nuclei, then the nucleus and the CXCR4 were respectively stained with 4΄,6-diamidino-2-phenylindole (DAPI) (blue) and with Cy2 (green). The photo was made by a Zeiss semi confocal epi-fluorescence microscope.

### Western blotting assay

SiRNA–CTL, siRNA–CXCR4-1, or siRNA–CXCR4-2 treated MCF‐7 cells were lysed with lysis buffer (Invitrogen, U.S.A.) on ice for 20 min, and the cell lysates were centrifuged at 13000 ***g*** at 4°C for 30 min, then the supernatant was collected as the total cellular protein extract. After determining protein concentration using the BCA Protein Assay Kit (Bio-RAD, U.S.A.), equal amount of each cellular protein was loaded onto 10% SDS polyacrylamide gel. The separated proteins were electrophoretically transferred to PVDF membranes (Bio-RAD, U.S.A.). The membrane was blocked overnight in blocking buffer containing PTST and 5% non-fat milk. Then, the membrane was incubated with primary mouse antibodies against CXCR4 and GAPDH for 1 h separately and was washed with PBST for four times subsequently. Following incubating with the secondary goat anti-mouse HRP-conjugated antibody for 1 h, the PVDF membrane was washed for four times and was treated with ECL reagent (Pierce, U.S.A.) and subjected to X-ray film. Each band was quantified using Image software.

### Quantitative real-time PCR assays for mRNA expression

Total RNA was extracted from the treated MCF‐7 cells using the RNeasy Mini-kit (Qiagen Inc.) according to the manufacturer’s recommendation and was reverse transcribed with SuperScript II RT (Invitrogen-Gibco). Quantitative real-time PCR was then conducted with SYBR® Green mastermix (Life tech, U.S.A.) in a 7500 Fast PCR instrument (Applied Biosystems, U.S.A.) using the special primers for CXCR4 mRNAs, separately. The cycle threshold (*C*_t_) values of the target gene were normalized to β-actin from the same sample as relative mRNA levels. All samples were run in triplicate in the 96-well reaction plates.

### Methyl thiazolyl tetrazolium assay

MCF‐7 cells were seeded into 96-well plates at a density of 1 × 10^4^ per well and incubated overnight in DMEM containing 10% heat-inactivated FBS. Cisplain was dissolved in DMSO and diluted with DMEM medium to final concentrations of 50 μM. The tumor cells were incubated with cisplain and were transfected with 0, 25, or 50 nM siRNA–CTL or siRNA–CXCR4 1 for 24 h before the methyl thiazolyl tetrazolium (MTT) assay. After removing the medium supernatant, 200 μl of DMSO was added into each well and mixed thoroughly. The plate was incubated at 37°C to dissolve air bubbles for 5 min, and *A*_570_ value of each well was measured at 570 nm wavelengths using a microplate reader (Thermo scientific, U.S.A.). The results were calculated as (*A*_570_ of control wells − *A*_570_ of treated wells)/(*A*_570_ of control wells − *A*_570_ of blank wells) × 100%.

### Cell counting assay and colony forming assay

For cell counting assay, MCF-7 cells (10^3^ per well) were treated with 50 μM cisplatin, and then were transfected with 50 nM siRNA–CTL or siRNA–CXCR4 1. After 1, 2, or 3 days, cells were washed two times with PBS, were detached with 0.25% trypsin, and then were counted. For the colony forming assay, MCF-7 cells were seeded in 12-well plate (200 cells per well) and were incubated in DMEM containing 10% FBS at 37°C. After separated colonies were formed (incubation for 5 days), cells were treated with cisplatin and were transfected with siRNA–CTL or siRNA–CXCR4 1. After another incubation (DMEM + 10% FBS) for 48 h, colonies were counted respectively.

### Statistical analysis

All experiments were assayed in triplicate. Data are expressed as means ± SD. All statistical analyses were performed using GraphPad Pro. Prism 5.0 (GraphPad, San Diego, CA, U.S.A.). Statistical differences between two groups were assessed by Student’s *t* test. *P* value <0.05 was considered statistically significant.

## Results

### Chitosan–siRNA–CXCR4 transfection efficiently down-regulated *CXCR4* expression in breast cancer MCF-7 cells

**Figure 1:**
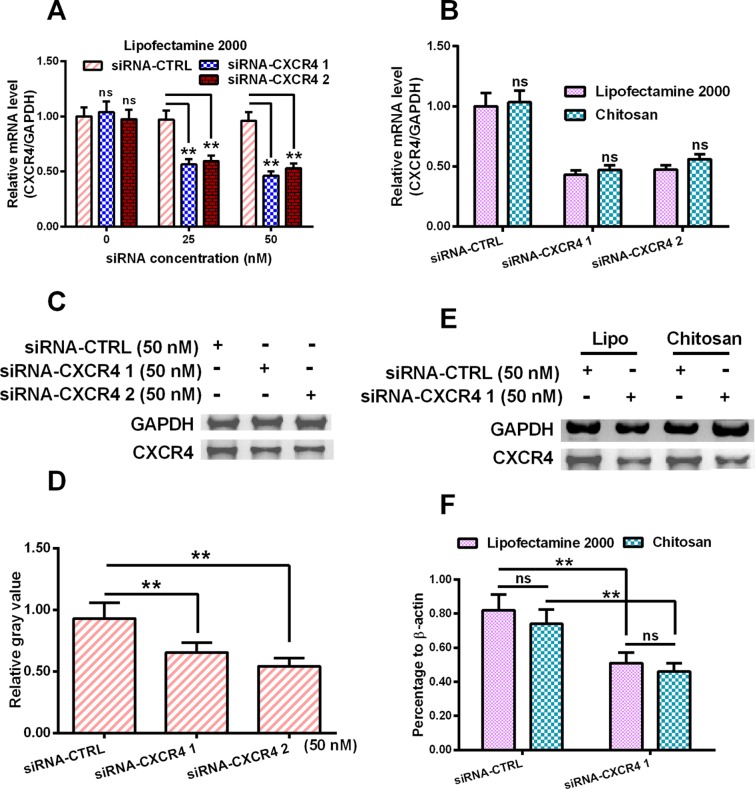
Regulation by chitosan–siRNA–CXCR4 on CXCR4 expression in breast cancer MCF-7 cells (**A**) CXCR4 mRNA level in the MCF-7 cells, which were transfected with 0, 25, or 50 nM CXCR4-specific siRNA–CXCR4 (siRNA–CXCR4 1 or siRNA–CXCR4 2) or control siRNA (siRNA–CTRL) for 12 h. (**B**) CXCR4 mRNA level in the MCF-7 cells, which were transfected by Lipofectamine 2000 or delivered by chitosan with siRNA–CXCR4 1/2- or siRNA–CTRL for 12 h. (**C**) and (**D**) Western blotting assay (C) and relative level (D) of CXCR4 (to GAPDH) in the MCF-7 cells, which were transfected with 50 nM siRNA–CXCR4 1, siRNA–CXCR4 2, or siRNA–CTRL for 24 h. (**E**) and (**F**) Western blotting assay (E) and relative level (F) of CXCR4 (to GAPDH) in the MCF-7 cells, in which 50 nM siRNA–CXCR4 1 or siRNA–CTRL was transfected by Lipofectamine 2000 or were delivered by chitosan for 24 h. Data were averaged for triple independents results. Statistical significance was presented as **P<0.01; ns, not significant.

As shown in Figure 1, the relative mRNA level of CXCR4 was down-regulated by 50% and 40% respectively, when treated by 25 nM CXCR4-specific siRNA (siRNA–CXCR4 1 or siRNA–CXCR4 2). Similarly, it was decreased by 60% and 48% separately, when treated by 50 nM siRNA–CXCR4 1 and siRNA–CXCR4 2 respectively. We then checked the transfection efficacies of Lipofectamine 2000 and chitosan respectively. At the mRNA level, no statistical difference was found between two groups, indicating those two transfection reagents have similar transfection efficiencies (Figure 1B). The *CXCR4* expression in protein level of treated MCF-7 cells was detected by WB. As shown in Figure 1(C) and (D), siRNA–CXCR4 1 or SiRNA–CXCR4 2 transfection obviously decreased the CXCR4 expression with statistical difference. The protein levels of CXCR4 and GAPDH were detected using WB after transfection of siRNA ctrol/siRNA–CXCR4 using Lipofectamine 2000 and chitosan separately. As indicated in Figure 1(E) and (F), siRNA–CXCR4 1 transfection efficiently decreased the CXCR4 expression by 37.5%, and chitosan transfection down-regulated the CXCR4 expression by 35%.

### Physical characteristics of chitosan–siRNA–CXCR4 nanoparticles

**Figure 2:**
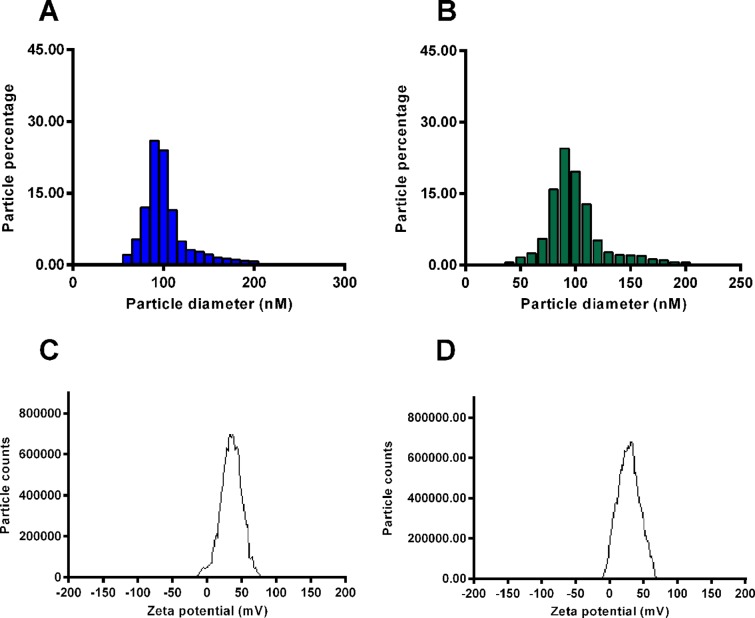
Diameter and zeta potential of chitosan–siRNA–CXCR4 particles The size (diameter in nm) of chitosan–siRNA–CXCR4 1 (particles coated by chitosan and siRNA–CXCR4) (**A**) and chitosan–miRNA–CTRL (particles coated by chitosan and control miRNA) (**B**) was analyzed by photon correlation spectroscopy. The zeta potential of chitosan–siRNA–CXCR4 1 (**C**) and chitosan–miRNA–CTRL (**D**) was measured by a Zetasizer Nano ZS.

**Figure 3:**
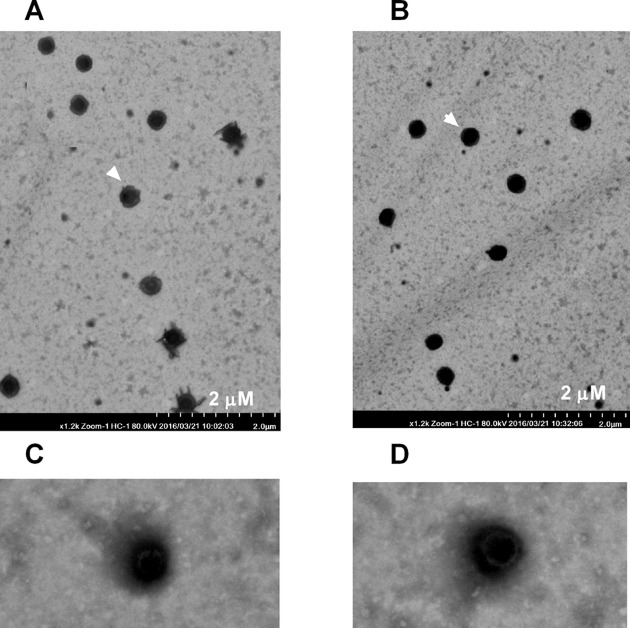
Image of chitosan–siRNA–CXCR4 particles under transmission electron microscopy (TEM) The particles of chitosan–siRNA–CXCR4 1 (**A**) and chitosan–miRNA–CTRL (**B**) were imaged under TEM at 5000×, with ruler indicated at bottom right; both particles (**C**: chitosan–siRNA–CXCR4, **D**: chitosan–miRNA–CTRL) were also imaged under 25000× magnifcation.

We measured the diameter and the zeta potential of chitosan–siRNA–CXCR4 particles. Particles sizes of chitosan–siRNA–CXCR4 1 and chitosan–miRNA–CTRL mainly focused at 100 nm (Figure 2A and B), and zeta potential of chitosan–siRNA–CXCR4 1 and chitosan–miRNA–CTRL is approximately ±70 mV, indicating good stability of those particles (Figure 2C and D). The particles of chitosan–siRNA–CXCR4 1 and chitosan–miRNA–CTRL were imaged under TEM at 5000×, with ruler indicated at bottom right (Figure 3A and B). Most of the particles diameters focused on the 200 nm (Figure 3C and D) under the 25000× magnification.

**Figure 4:**
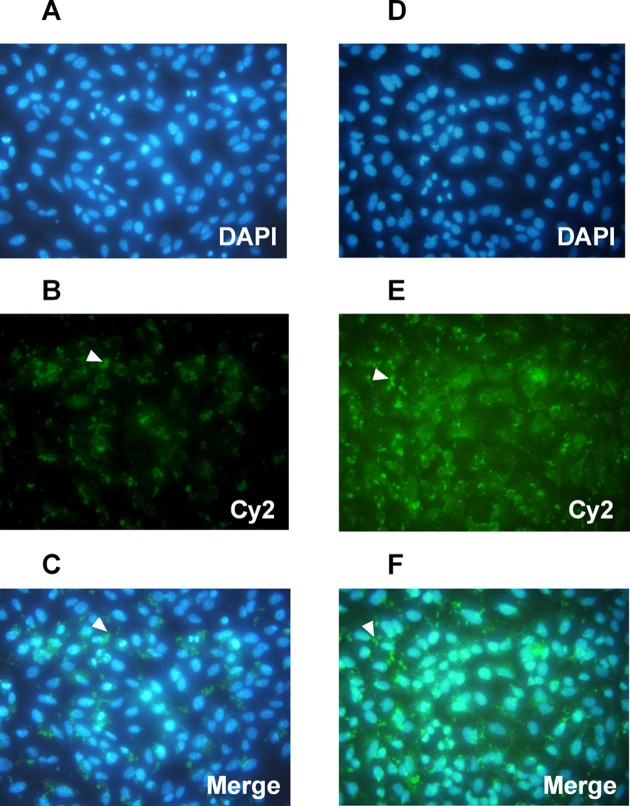
Representative confocal scanning laser microscopy images of CXCR4 expression in MCF-7 cells post the chitosan–siRNA–CXCR4 delivery siRNA–CXCR4 1 and siRNA–CTRL were delivered into 85% confluent MCF-7 cells with chitosan for 24 h, then the nucleus and the CXCR4 were respectively stained with 4΄,6-diamidino-2-phenylindole (DAPI) (blue) and with Cy2 (green). (**A**–**C**) Representative confocal scanning for nucleus (A), CXCR4 (B), or merged image (C) for siRNA–CXCR4 1-delivered cells; (**D**–**F**) Representative confocal scanning for nucleus (D), CXCR4 (E), or merged image (F) for siRNA–CTRL-delivered cells.

To reconfirm the delivery efficiency of siRNA–CXCR4 by chitosan, we then performed the confocal scanning laser microscopy images of CXCR4 expression in MCF-7 cells post the chitosan–siRNA–CXCR4 delivery. After siRNA–CXCR4 1 and siRNA–CTRL were delivered into 85% confluent MCF-7 cells with chitosan for 24 h, then the nucleus and the CXCR4 were respectively stained with 4΄,6-diamidino-2-phenylindole (DAPI) (blue) and with Cy2 (green) for both siRNA–CXCR4 1 (Figure 4A–C) and siRNA–CTRL (Figure 4D–F). It was clearly indicated that the CXCR4 staining was reduced in the chitosan–siRNA–CXCR4 1-delivered cells (Figure 4A–C), compared with the chitosan–siRNA–CTRL-delivered cells (Figure 4D–F).

### Chitosan–siRNA–CXCR4 sensitizes breast cancer MCF-7 cells to cisplatin

**Figure 5:**
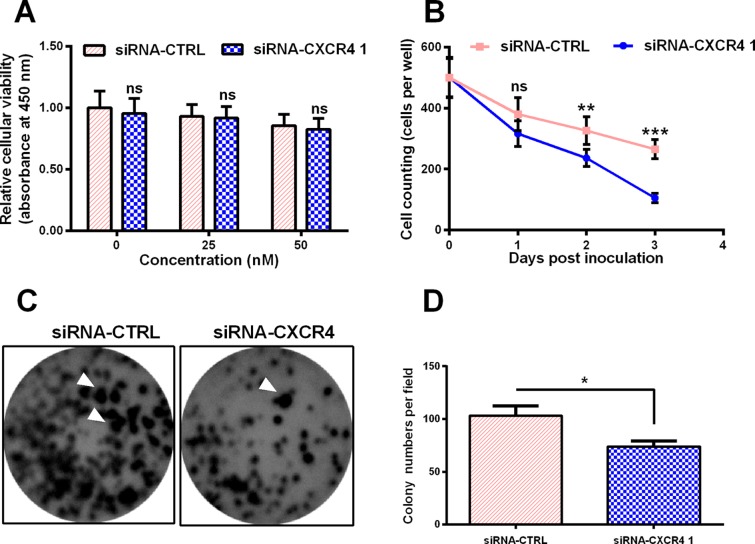
chitosan–siRNA–CXCR4 sensitizes breast cancer MCF-7 cells to cisplatin *in vitro* (**A**) MTT assay for the viability of the MCF-7 cells post the chitosan-mediated delivery of siRNA–CXCR4 1 or siRNA–CTRL (0, 25, or 50 nM) for 24 h. (**B**) Growth curve of the MCF-7 cells post the chitosan-mediated delivery of siRNA–CXCR4 1 or siRNA–CTRL, in the presence of 50 μM cisplain. Cells were titered at the 0, 1, 2, or 3 day post siRNA delivery. (**C**) and (**D**) Images (C) and counting (D) of colonies formed by the MCF-7 cells post the chitosan-mediated delivery of siRNA–CXCR4 1 or siRNA–CTRL (50 nM) for 48 h, in the presence of 50 μM cisplain. The experiments were performed independently in triplicate. Statistical significance was shown as *P<0.05, **P<0.01, ***<0.001, ns, not significant.

We measured the viability of the MCF-7 cells post the chitosan-mediated delivery of siRNA–CXCR4 1 or siRNA–CTRL (0, 25, or 50 nM) for 24 h, and no statistical differences were found between groups (Figure 5A and B). The result indicated that chitosan-mediated delivery of siRNA–CXCR4 1 or siRNA–CTRL had no effect on cellular viability. In the next step, we mapped the growth curve of the MCF-7 cells post the chitosan-mediated delivery of siRNA–CXCR4 1 or siRNA–CTRL, in the presence of 50 μM mg/ml cisplain. We found siRNA–CXCR4 1 decreased the cell counting per well by 25% (*P*<0.001) and 50% (*P*<0.001) respectively, at day 2 or 3 post-infection. We tested the colonies formed by the MCF-7 cells post the chitosan-mediated delivery of siRNA–CXCR4 1 or siRNA–CTRL, in the presence of 50 μM cisplain. As shown in Figure 5(C) and (D), the SiRNA–CXCR4-treated group clearly down-regulated the colony numbers by 30% (*P*<0.05) as compared with the siRNA–control group.

## Discussion

In the present study, we investigated the delivery efficiency of siRNA by chitosan into breast cancer cells, and examined the regulatory role by chitosan nanoparticle-delivered siRNA on CXCR4 expression and on the chemosensitivity of breast cancer cells. We found the siRNA could be efficiently capsuled by chitosan into nanoparticles, and that delivered siRNA efficiently reduced the expression of *CXCR4* in both mRNA and protein levels. Moreover, the reduced *CXCR4* expression was associated with increased sensitivity of breast cancer cells to cisplatin. Our results present the treatment potential of chitosan nanoparticle-delivered siRNA targeting *CXCR4* in breast cancers.

Several reports have reported that chemokines and their receptors play critical roles in the development and progression of cancer. In all the known chemokine receptors, breast cancer cells specifically express active CXCR4, which was associated with metastatic breast cancer. Therefore, novel drugs capable of decreasing the CXCR4 level may be a potential therapy for breast cancer treatment. Here, we found that the reduced CXCR4 by chitosan nanoparticle-delivered siRNA was associated with increased sensitivity of breast cancer cells to cisplatin. The result is in accordance with the previous report [17]. However, the clinical application of this strategy still needs more supported *in vivo* experiments, because of the side effects from concomitant mobilization of bone marrow stem cells, particularly, CXCR4 delivery technique might affect hematopoietic and progenitor cells in the bone marrow [18].

General strategy for cancer treatment is to use multiple drugs targeting different molecular targets. Decreasing the levels of target proteins implied in signaling pathways of tumor cell survival or proliferation using the siRNA technology has increased [19]. Viral vectors were initially used, but high cost and the host immune response have limited its use in animals [20]. Novel vectors such as nanoparticles have been proposed as a good tool for delivering siRNA to different cell types [21]. In our study, we found that the siRNA could be efficiently capsuled by chitosan into nanoparticles with a diameter of 80–110 nm and with a zeta potential of 20–50 mV. The chitosan nanoparticle delivered siRNA efficiently into MCF-7 cells and significantly reduced the expression of CXCR4 in both mRNA and protein levels. Our results indicate that chitosan nanoparticle is an ideal tool to deliver siRNA into target cells.

In conclusion, our results present the treatment potential of chitosan nanoparticle-delivered siRNA targeting CXCR4 in breast cancers. Further research on animal experiments will be performed in the near future.

## Compliance with Ethical Standards

## Abbreviations

BRCA1/2: breast cancer associated gene 1/2
CTL: cytotoxic T lymphocyte
CXCR4: chemokine receptor 4
Cy5: cyanine dye 5
DMEM: Dulbecco's Modified Eagle's Medium
GAPD: glyceraldehyde 3-phosphate dehydrogenase
HRP: horseradish peroxidase
MCF-7: A human breast adenocarcinoma cell line
PTST: phosphate Buffered Saline with Tween 20
SDF-1: stromal cell-derived factor 1
WB: western blottin
