# Expression of Concern: Chitosan nanoparticle-delivered siRNA reduces CXCR4 expression and sensitizes breast cancer cells to cisplatin

**DOI:** 10.1042/BSR20170122_EOC

**Published:** 2026-07-24

**Authors:** Shaonan Yu, Yan Chen, Xuefeng Li, Zhongli Gao, Guifeng Liu

**Affiliations:** 1 Department of Orthopedics, China-Japan Union Hospital of Jilin University, 130033 Changchun, China; 2 Department of Radiology, China-Japan Union Hospital of Jilin University, 130033 Changchun, China; 3 Department of Endocrinology, Second Hospital of Jilin University, 130041 Changchun, China; 4 Department of Anesthesiology, China-Japan Union Hospital of Jilin University, 130033 Changchun, China

**Keywords:** Breast cancer cells, Chitosan, Cisplatin, CXCR4, siRNA

*Bioscience Reports* has been made aware of potential issues surrounding the scientific validity of this paper and is hence issuing a permanent Expression of Concern to notify readers.

Specifically, it was noted that the second and third Figure 1C CXCR4 bands are highly similar to the first two Figure 5B TLR-4 bands of the article Fang and Jiang, 2016 (DOI: 10.1042/BSR20160118). The authors provided raw data but concerns remain.

As such, the article will remain published but with an associated Expression of Concern.

